# The Roads to Mitochondrial Dysfunction

**DOI:** 10.1155/2015/235370

**Published:** 2015-01-13

**Authors:** Marcos Roberto de Oliveira, Namasivayam Elangovan, Marko Ljubkovic, Ancha Baranova

**Affiliations:** ^1^Department of Chemistry, Postgraduate Program of Chemistry, Federal University of Mato Grosso, 78060-900 Cuiabá, MT, Brazil; ^2^Molecular Pharmacology Research Laboratory, Department of Biotechnology, Periyar University, Salem, India; ^3^Department of Physiology, University of Split School of Medicine, Soltanska 2, 21000 Split, Croatia; ^4^Center for the Study of Chronic Metabolic Diseases, School of System Biology, George Mason University, Fairfax, VA 22030, USA; ^5^Research Center for Medical Genetics RAMS, Moskvorechie Street, 1, Moscow, Russia; ^6^Institute of Physics and Technology, Institutsky 9, Dolgoprudny 141700, Russia

Mitochondria are the major site of ATP production in animal cells. The proton gradient obtained via electron transport and proton pumping in the electron transport chain is utilized by complex V to produce ATP. However, this elaborated system is prone to the electron leak that serves as the main source of superoxide anion radical (O_2_^−∙^). Additionally, other reactive species and free radicals may be produced through reactions catalyzed by antioxidant enzymes, such as superoxide dismutase, which produces H_2_O_2_ from O_2_^−∙^. An intrinsic way that leads to mitochondrial dysfunction is an increased production of reactive oxygen or nitrogen species and free radicals within this organelle. Also, other stimuli, such as presence of toxicants, protein aggregates, and stress on other organelles (as, for instance, endoplasmic reticulum stress), affect mitochondrial function and may induce mitochondrial swelling and/or mitochondrial permeability transition pore formation, which is an initial step to apoptosis. In fact, mitochondrial dysfunction at high rates is the major cause of cell death in a myriad of diseases and during aging.

In this special issue, the details of the dysfunction in electron transport chain and/or respiration are analyzed in three studies. V. Jeger et al. looked at the effects of an exposure to bacterial endotoxin, also known as lipopolysaccharide in hepatic cell line HepG2 and in primary hepatocytes, as well as on mitochondria isolated from quadriceps muscle of pigs. Interestingly, the effects of LPS varied depending on the biological system analyzed. In any case, it seems that LPS is capable of affecting mitochondrial function through altering either mitochondrial membrane potential or respiration. T. Boczek et al. studied the effects of silencing plasma membrane Ca^2+^-ATPase isoforms 2 and 3 on energy metabolism in PC12 cells during differentiation. The researchers found that alterations in the amounts of these types of the protein pump perturb energy-generating pathways and mitochondrial activity. The researchers found that alterations in the level of such protein pump altered energy-generating pathways in order to maintain ATP homeostasis. This may be linked to the role of such proteins in controlling Ca^2+^ ions concentration within a physiological range during neuronal differentiation. J. R. Macarini et al. investigated the effects of carnosine on mitochondrial function isolated from rat skeletal muscle. They found that only acute carnosine exposition altered mitochondrial function. This is an important finding since there is accumulation of carnosine in carnosinase deficiency, a disease that affects several human tissues.

Z. Hu and J. Tu investigated mitochondrial function in an experimental model of posttraumatic syringomyelia. The researchers found that the decrease in mitochondrial ATP outputs and other metabolic scores associated with the degree of mitochondrial impairment are the primary contributors to glutamate neurotoxicity that, in turn, may lead to necrosis and enlargement of the cavity in the model of posttraumatic syringomyelia. Importantly, if the mitochondrial damage was reversible, as it was observed in mechanical laminectomy model, the neuronal cell death was reversible as well. This observation explains why no cavity forms after laminectomy and places mitochondrial dysfunction at the heart of the posttraumatic neuronal recovery.

In their review paper, J. Su et al. analyzed the crosstalk between endoplasmic reticulum stress and mitochondrial dysfunction in tumors during the development of chemotherapy resistance and provided systematic evidence that this crosstalk is mediated by activities of Bcl-2 family of the proteins.

Two papers of this special issue deal with the variation of mitochondrial function in human populations. D. A. Chistiakov et al. performed a thorough review of mitochondrial aging and mitochondrial dysfunction in elderly age and the strategies of attenuating the age-related phenotype in humans, while I. A. Sobenin et al. reviewed techniques that help to reliably investigate levels of heteroplasmy of mitochondrial mutations in a variety of diseases, including those associated with aging.

Finally, A. Panov et al. point that the neuronal mitochondria that are capable of simultaneous utilization of several substrates, including various neuromediators, in particular, glutamate and GABA, supplied neurons with astroglia. Authors reconcile *β*-oxidation of fatty acids by astrocytic mitochondria with the regulation of aerobic glycolysis and provided experimental evidence that isolated neuronal mitochondria may oxidize palmitoyl carnitine in the presence of other mitochondrial substrates.

Altogether, this eclectic collection of papers reflects various aspects of the current understanding of the interplay between the functioning of the mitochondrial genome, the components of respiratory chain it encodes, and the mutual influences of the mitochondria and the cell and discusses potential impact of the mitochondrial dysfunction on some health conditions. There is a hope that an increase in understanding of molecular mechanisms of mitochondrial dynamics may lead to new therapeutic approaches for the prevention or amelioration of the diseases associated with ailing mitochondria.

*Marcos Roberto de Oliveira* *Namasivayam Elangovan* *Marko Ljubkovic* *Ancha Baranova*

